# Characterizing a Foraging Hotspot for Short-Finned Pilot Whales and Blainville’s Beaked Whales Located off the West Side of Hawai‘i Island by Using Tagging and Oceanographic Data

**DOI:** 10.1371/journal.pone.0142628

**Published:** 2015-11-25

**Authors:** Melanie Abecassis, Jeffrey Polovina, Robin W. Baird, Adrienne Copeland, Jeffrey C. Drazen, Reka Domokos, Erin Oleson, Yanli Jia, Gregory S. Schorr, Daniel L. Webster, Russel D. Andrews

**Affiliations:** 1 Joint Institute for Marine and Atmospheric Research, University of Hawai’i, Honolulu, HI, United States of America; 2 Pacific Islands Fisheries Science Center, NOAA Fisheries, Honolulu, HI, United States of America; 3 Cascadia Research Collective, Olympia, WA, United States of America; 4 Hawai’i Institute of Marine Biology, University of Hawai’i, Kāne’ohe, HI, United States of America; 5 Department of Oceanography, University of Hawai’i, Honolulu, HI, United States of America; 6 International Pacific Research Center, School of Ocean and Earth Science and Technology, University of Hawai’i, Honolulu, HI, United States of America; 7 Alaska SeaLife Center, Seward, AK, United States of America; 8 School of Fisheries and Ocean Sciences, University of Alaska Fairbanks, Fairbanks, AK, United States of America; UC Santa Cruz Department of Ecology and Evolutionary Biology, UNITED STATES

## Abstract

Satellite tagging data for short-finned pilot whales (*Globicephala macrorhynchus*) and Blainville’s beaked whales (*Mesoplodon densirostris*) were used to identify core insular foraging regions off the Kona (west) Coast of Hawai‘i Island. Ship-based active acoustic surveys and oceanographic model output were used in generalized additive models (GAMs) and mixed models to characterize the oceanography of these regions and to examine relationships between whale density and the environment. The regions of highest density for pilot whales and Blainville’s beaked whales were located between the 1000 and 2500 m isobaths and the 250 and 2000 m isobaths, respectively. Both species were associated with slope waters, but given the topography of the area, the horizontal distribution of beaked whales was narrower and located in shallower waters than that of pilot whales. The key oceanographic parameters characterizing the foraging regions were bathymetry, temperature at depth, and a high density of midwater micronekton scattering at 70 kHz in 400–650 m depths that likely represent the island-associated deep mesopelagic boundary community and serve as prey for the prey of the whales. Thus, our results suggest that off the Kona Coast, and potentially around other main Hawaiian Islands, the deep mesopelagic boundary community is key to a food web that supports insular cetacean populations.

## Introduction

The west coast of the Island of Hawai‘i Island, also called the Kona Coast, supports a number of commercial [1], recreational, and subsistence fisheries and is the site of an annual international billfish tournament [2], open-ocean aquaculture projects and numerous ocean activities catering to tourists and residents. It is also home to more than a dozen species of cetaceans [3–5], which are federally protected under the Marine Mammal Protection Act (MMPA). Having a good understanding of the areas occupied by each species and being able to define regions of essential habitat is key for environmental planning to mitigate potential impacts of anthropogenic activities on marine mammal populations in the area.

Reliable assessments of range and habitat utilization are challenging for highly mobile and long-diving cetacean species. Visual surveys are associated with important biases [4,6]. Further, for difficult-to-detect species, such as beaked whales, obtaining large sample sizes from visual surveys is difficult. When available, satellite tagging data offer a more complete picture of animal movements and range and allow species-specific relationships between environmental factors and habitat preferences to be studied more rigorously [7–10]. Additionally, habitat modeling can allow managers to predict the habitat use of a species on a finer scale than that typically provided by visual transect surveys [11].

A collaborative effort including Cascadia Research Collective, Alaska SeaLife Center, Northwest Fisheries Science Center, and Pacific Islands Fisheries Science Center has been engaged in satellite tagging of cetaceans off the Kona Coast since 2006. This paper focuses on two species of odontocetes whose habitat preferences are poorly understood and for which sufficient tagging data were available–short-finned pilot whales (*Globicephala macrorhynchus*) and Blainville’s beaked whales (*Mesoplodon densirostris*).

Both species are found worldwide in tropical and temperate waters. For both species, there is evidence for the existence of an island-associated population in the main Hawaiian Islands [12–15] as well as a core resident population off Hawai‘i Island for short-finned pilot whales [16]. Pilot whales have been shown to dive down to 1300 m (R. Baird, pers. comm.), with a maximum dive duration of 27 mins in the Hawai‘ian Archipelago [17] and 1000 m (maximum dive duration of 21 mins) in the Canary Islands [18]. In both locations, the deeper dives (> 500 m) occurred primarily during the daytime. In the Canary Islands, such dives are typically associated with sprinting events coinciding with a foraging buzz at the deepest point of the dive [18]. Blainville’s beaked whales in Hawai‘i have been shown to dive as deep as 1600 m (maximum dive duration of 83 mins) [19], with deep foraging dives (> 800 m) occurring at the same rate during daytime and night-time and midwater dives (between 100 and 600 m) occurring primarily during the daytime [20]. Similarly beaked whales in the Canary Islands show no circadian change in foraging [21]. Both species have a reduced dentition (arrangement of the teeth) adapted to suction-feeding on cephalopods [22]. Little is known about the diet of these species, but likely fish and mesopelagic squid [23] are both consumed among other prey.

Here we use data from satellite tags deployed on both species in waters off the Kona Coast of Hawai‘i Island as well as active acoustic data collected during research cruises and data from a high-resolution ocean general circulation model to assess factors determining the areas of highest use for these species located off the Kona Coast.

## Methods

### Satellite tagging

Multi-species cetacean field studies have been conducted off the west side of the Island of Hawai‘i from 2002 through 2011 by Cascadia Research Collective [4,15,20,24]. In this study we utilized location data from satellite tag data for short-finned pilot whales (n = 46) and Blainville’s beaked whales (n = 12) from 2006 through 2011 (Table 1). Tagging was conducted under Scientific Research Permit Nos. 782–1789, 731–1774 and 15330 issued by the National Marine Fisheries Service. Satellite tags used were Wildlife Computers (Redmond, WA) location-only SPOT5 tags (n = 12) for beaked whales and both SPOT5 (n = 37) and depth-transmitting Mk10-A tags (n = 9) for pilot whales.

**Table 1:** Summary of animals satellite tagged.

Species	Whale ID	Deployment Date	End Date	Duration (# days)
Pilot whale	HIGm0384	11/21/06	12/08/06	16.47
Pilot whale	HIGm0703	08/11/07	08/21/07	9.46
Pilot whale	HIGm0723	08/11/07	08/14/07	2.44
Pilot whale	HIGm0309	08/13/07	08/21/07	7.51
Pilot whale	HIGm0353	04/20/08	05/14/08	23.17
Pilot whale	HIGm0346	05/14/08	07/05/08	52.04
Pilot whale	HIGm0427	07/03/08	09/13/08	72.02
Pilot whale	HIGm1034	07/03/08	07/18/08	14.53
Pilot whale	HIGm0351	07/03/08	08/30/08	57.98
Pilot whale	HIGm0352	07/03/08	08/19/08	46.12
Pilot whale	HIGm0680	07/05/08	08/04/08	30.96
Pilot whale	HIGm0471	07/05/08	08/02/08	28.83
Pilot whale	HIGm0704	07/05/08	08/12/08	37.53
Pilot whale	HIGm0712	07/05/08	08/07/08	32.3
Pilot whale	HIGm0715	07/05/08	07/19/08	13.79
Pilot whale	HIGm0726	07/08/08	07/29/08	20.4
Pilot whale	HIGm0446	07/10/08	08/19/08	39.98
Pilot whale	HIGm0840	07/12/08	09/16/08	65.96
Pilot whale	HIGm0190	07/13/08	08/13/08	30.76
Pilot whale	HIGm0733	07/19/08	09/23/08	65.19
Pilot whale	HIGm0839	07/25/08	08/06/08	11.84
Pilot whale	HIGm1420	04/30/09	06/07/09	38.5
Pilot whale	HIGm0184	04/30/09	05/25/09	25.43
Pilot whale	HIGm1036	04/30/09	07/25/09	86.19
Pilot whale	HIGm0229	05/02/09	08/20/09	109.91
Pilot whale	HIGm0153	10/25/09	12/07/09	42.5
Pilot whale	HIGm0427	10/25/09	11/25/09	30.12
Pilot whale	HIGm0745	10/31/09	12/10/09	39.77
Pilot whale	HIGm0343	12/15/09	01/31/10	47.07
Pilot whale	HIGm0565	12/15/09	01/04/10	19.7
Pilot whale	HIGm0296	12/17/09	12/22/09	4.55
Pilot whale	HIGm0224	12/17/09	01/28/10	42.09
Pilot whale	HIGm0840	12/18/09	01/04/10	16.38
Pilot whale	HIGm0386	12/13/10	01/18/11	36.67
Pilot whale	HIGm0841	12/16/10	01/19/11	33.91
Beaked whale	HIMd118	11/22/06	12/15/06	22.73
Beaked whale	HIMd120	11/23/06	12/09/06	16.18
Beaked whale	HIMd001	12/04/06	12/19/06	14.76
Beaked whale	HIMd025	07/10/08	09/11/08	62.57
Beaked whale	HIMd020	07/10/08	08/31/08	51.25
Beaked whale	HIMd007	07/10/08	09/11/08	63.22
Beaked whale	HIMd148	07/14/08	09/23/08	71.24
Beaked whale	HIMd036	07/14/08	08/28/08	44.97
Beaked whale	HIMd153	04/29/09	06/08/09	39.47
Beaked whale	HIMd168	12/21/09	01/11/10	20.51
Beaked whale	HIMd121	10/23/11	12/06/11	43.12
Beaked whale	HIMd014	05/09/12	07/22/12	74.13

### Ethics Statement

All tagging was undertaken in Hawai‘i waters by Cascadia Research Collective in full compliance of Scientific Research Permit No. 782–1789, 731–1774 and 15330. All permits were issued by the U.S. National Marine Fisheries Service (NMFS).

Tag dimensions were 65 × 30 × 22 mm. Each tag incorporated two 6.5 cm long medical-grade titanium darts that were screwed into 2 holes in the bottom of the tag. The darts were designed to penetrate the connective tissue in the dorsal fin and remain embedded with a series of backwards-facing ‘barbs’, which acted as anchors for the darts [25]. The weight of the entire package was approximately 49 g. The transmitter electronics unit was designed to remain external to the body to minimize the invasiveness of the technique. Tags were deployed using a Dan-Inject JM Special 25 (Børkop, Denmark) pneumatic projector, with a modified arrow to hold the tag in flight at a range of 3 to 10 m [14].

Prior to 2010, all tagging work was approved by the Alaska SeaLife Center’s Institutional Animal Care and Use Committee (IACUC). All subsequent tagging work was approved by Casacadia Research Collective's IACUC.

All tagging/field procedures were reviewed and approved as part of the NMFS Scientific Research Permits, which include authorization to work in all Hawaiian waters.

Tags were set to transmit for varying numbers of hours throughout the day generally corresponding with periods of maximum satellite coverage. Position estimates of tagged whales were made by the Argos System using the least-squares method, and were processed with the Douglas Argos filter [26], ver. 7.08 to eliminate unrealistic position estimates using the filtering parameters outlined in Baird et al. [25].

To further filter out Argos location errors, all tracks were processed in a similar way as in Gaspar et al. [27] and Abecassis et al. [8]. First, all locations resulting from velocities greater than 20 km/h were removed. Then, to remove additional artificial ‘‘spikes” from the data, an Epanechnikov filter [28] was applied with a 12-hour window, centered on each individual location, using the *lpepa* function of the *lpridge* package [29] in the R environment [30]. The points providing the 5% largest differences between the Douglas-filtered and Epanechnikov-filtered locations were removed. The Douglas-filtered track without these extreme locations was then resampled using the *lpepa* function with a one/hour window from a few locations a day (average number of locations = 8.3/day and 4.5/day for pilot and beaked whales respectively) to one location per hour.

### Kernel densities

Kernel density estimates of whale space use were computed from the filtered and interpolated tag position estimate data (all years combined) using the *kde2d* function of the *MASS* package [31] in R over the area 19.2–20.1°N, 155.9–156.7°W, which encompasses all animal tracks and cruise transects (see below), on a 0.01° × 0.01° grid. The bandwidths of the Gaussian kernel density estimator were selected using the *width*.*SJ* function [32]. Densities are expressed in total hours (over all individuals of each species) spent per 0.01° × 0.01° grid cell.

### Acoustic data

Short-finned pilot whales and Blainville’s beaked whales feed on squids and fishes that in turn prey on micronektonic organisms [33]. We are unable to directly sample the elusive prey of the cetaceans but are able to sample the community believed to serve as prey for the cetaceans’ prey using active acoustics. Relative micronekton density was estimated from in situ acoustic backscatter data collected continuously on board the NOAA R/V *Oscar Elton Sette* during 3 cruises: July 2011, June 2013, and March 2014. The ship is equipped with a hull-mounted Simrad EK60 split-beam echosounder system operating at 38 kHz and 70 kHz frequencies. The 38 kHz frequency allows us to sample the whole deep scattering layer (> 1000 m), whereas the 70 kHz frequency, while able to sample smaller organisms than the 38 kHz, is limited to about 670 m. The system was calibrated prior to the cruise in 2011 and 2014, and after the cruise in 2013 using a 38.1-mm-diameter tungsten carbide sphere according to standard methods [34]. During the July 2011 cruise, all transducers (~ 7° beam-widths) were set to operate with a 1024 μs pulse length. During the June 2013 and March 2014 cruises the pulse length was reduced to 512 μs to increase resolution. Power for the 38 kHz and 70 kHz frequencies was set at 2 kW and 0.75 kW, respectively.

To avoid backscatter from plankton and other smaller organisms, the minimum threshold for the mean volume backscattering strengths (Sv), a proxy for relative density [35], was set to—80 dB. Noise and bubble dropout were removed using ECHOVIEW 5.4 [36] and standard cleaning techniques [37]. Sv was binned in 100-m horizontal resolution and 10-m vertical resolution down to 1200 m and 670 m for 38 kHz and 70 kHz frequencies, respectively. Each cell was spatially integrated to obtain the relative acoustic biomass or nautical area-scattering coefficients (NASC) [35,38] in m^2^ nmi^-2^, which is used as a proxy for micronektonic organisms density, assuming that the composition of scatterers did not change significantly in space or time. NASC values from each of the three cruises were standardized by subtracting the mean value and then dividing the difference by the standard deviation to account for differences between years and seasons. The standardized NASC values from all three cruises were then combined to make a climatological field of micronekton density. We used generalized additive models (GAMs; [39]) with latitude and longitude as covariates to identify spatial patterns in NASC. All GAMs were computed in R using the *gam* function from the *mgcv* package [40]. We used the *predict*.*gam* function to extrapolate micronekton density over the whole study area. After examination of depth profiles of NASC, the data were separated into shallow (0–250 m) and deep (375–725 m for the 38 kHz and 375–665 m for the 70 kHz) components. Daytime and night-time NASC were examined separately, thus leading to 8 different micronekton components (one shallow and one deep component per day/night for each frequency, Table 2). The daytime and night-time acoustics transects were separated by identifying the start and end of the diurnal deep scattering layer migration to the surface. Night-time was defined as the time after sunset when the organisms started migrating to the surface. Daytime was defined as the time after sunrise when the organisms were back at depth.

**Table 2:** Summary of all covariates considered in the whale GAMMs: bathymetry, NASC values for 8 components of the micronekton from cruise data, currents absolute and vertical velocities, temperature and salinity at 11 depth levels from a general circulation model.

Bathymetry	NASC	Currents velocity (m)	Currents vertical velocity (m)	Temperature (m)	Salinity (m)
	70 kHz day shallow	0	0	0	0
	70 kHz day deep	100	100	100	100
	70 kHz night shallow	200	200	200	200
	70 kHz night deep	300	300	300	300
	38 kHz day shallow	400	400	400	400
	38 kHz day deep	500	500	500	500
	38 kHz night shallow	600	600	600	600
	38 kHz night deep	700	700	700	700
		800	800	800	800
		900	900	900	900
		1000	1000	1000	1000

### Trawling data

Micronektonic organisms were collected at six stations (3 offshore, 3 inshore) during the 2013 cruise using a dual warp modified Cobb trawl [41]. The open-mouth area was approximately 140 m^2^ with a mesh size of 152 mm stretched at the mouth to a cod end lined with 3.2-mm knotless nylon delta mesh netting. We conducted 2 night-time oblique trawls at each of the 6 stations, one at 21:00 and one at 01:00 local time. The deepest depths reached by the trawl ranged between 165 m and 257 m. Trawl depths were selected based on concurrently conducted acoustic surveys indicating the depths showing the greatest density of sound-scattering organisms. We fished each trawl for 60 min at a speed of 3 knots. To determine and record the depths fished, a Northstar Electronics Netmind trawl monitoring system was used. The Netmind was attached to the port wing of the trawl and sent data to the ship via acoustic telemetry on latitude, longitude, temperature, and depth. Two small TDRs (time-depth recorder) were attached to the head and foot ropes of the net in addition to the Netmind. A quick analysis of the collected samples was performed to check for the presence of species indicative of the mesopelagic boundary layer [42]: caridean shrimps and enoploteuthid squids from the collected samples were examined to estimate the abundance of *Oplophorus gracilorostris*, and *Abralia trigonura*, respectively.

### Modeling the distribution of whales

Because observations belonging to the same track cannot be considered independent of each other, generalized additive mixed effects models (GAMMs) were used to model the relationships between whale density and environmental covariates and micronekton density. All GAMMs were computed using the *gamm* function of the *mgcv* package [40]. Environmental variables considered were the following (Table 2): bathymetry from the School of Ocean and Earth Science and Technology of the University of Hawai‘i Main Hawaiian Islands Multibeam Bathymetry Synthesis [43] available at a 50-m spatial resolution; and zonal, meridional and vertical components of the current velocities, temperature, and salinity at various depths from a regional implementation of the Massachusetts Institute of Technology general circulation model (MITgcm) at a 1-km spatial resolution [44]. The MITgcm model simulation was run from April 1, 2011 to July 30, 2013, with output saved for each model day. The output includes 35 vertical layers, from the surface to 989 m depth. The regional MITgcm simulation does not assimilate observational (in situ or satellite) data. Model variables are initialized with the output of an ocean model simulation at a horizontal resolution of 0.04° (~ 4 km) configured for the main Hawaiian Islands [45] based on the Hybrid Coordinate Ocean Model (HYCOM) [46–48]. The HYCOM output is also used to define the open boundary conditions of the MITgcm. The simulation is designed to produce the observed characteristics of the ocean in a statistical sense, but may not coincide with observations on any given time step. For the purpose of this paper, we used the average of each variable of the MITgcm over the whole time range of the simulation in the GAMMs.

We rounded the kernel density estimates (response variable) to 2 decimal places and multiplied by 100 to obtain “pseudo-count” data for the response variable. Because these populations of whales are island-associated [4,14], the data contain a very high abundance of near-zero values offshore of the core distribution of each species, so we replaced all density values smaller than 0.12 (0.25) for pilot whales (beaked whales) with NaNs (Not a Number). Those thresholds were picked visually to minimize the presence of off-shore near-zero values in the data due to rare off-shore excursions while preserving the entirety of the core distribution of whales. We used a negative binomial distribution with a logarithmic link function to evaluate whale density as a function of the environmental and micronekton variables. Individual tag ID was used as a random effect in the GAMMs to account for autocorrelation within each track. Deviance explained was calculated as the ratio of the deviance of the full model compared to the deviance of the null model.

Potential confounding between statistically significant covariates was investigated with variance inflation factors (VIF) [49–51] using the *corvif* function available at http://www.highstat.com/book2.htm [50,51]. Model selection was performed manually, one variable at a time, and we retained candidate predictors that were statistically significant at the 0.05 level, maximized the amount of deviance explained and exhibited a VIF lower than 3. Once the final model was chosen, the number of knots (*k*) was sometimes manually decreased based on the appearance of the smooth plots, to further reduce overfitting. Model predictions were made via the *predict*.*gam* function of the *mgcv* package.

Although there was a lack of agreement in the temporal coverage of each of the data sets used in this study, we leveraged existing data sets as climatologies (averages over the temporal coverage of each data set) to characterize the average situation and linkages in the Kona Coast area. To examine the robustness of the results, the model was rerun using only 2011 data for pilot whales, as it is the year where all data sets overlap. This was not possible for beaked whales however, as only one tag was released in 2011 and the animal’s movement was not representative of the overall beaked whale distribution (S1 File).

To evaluate the potential trophic connection between whales and micronekton, a “trophic-only” single-variable GAMM was built for each cetacean species, with whale density estimates as the response variable and the micronekton component that explains the maximum amount of deviance as covariate.

Finally, to further examine the link between micronekton and whale distributions, a single-variable GAMM for whales and a single-variable GAM for the 4 deep micronekton components with bathymetry as the explanatory variable were used to investigate the diurnal dynamics.

## Results

**Fig 1:**
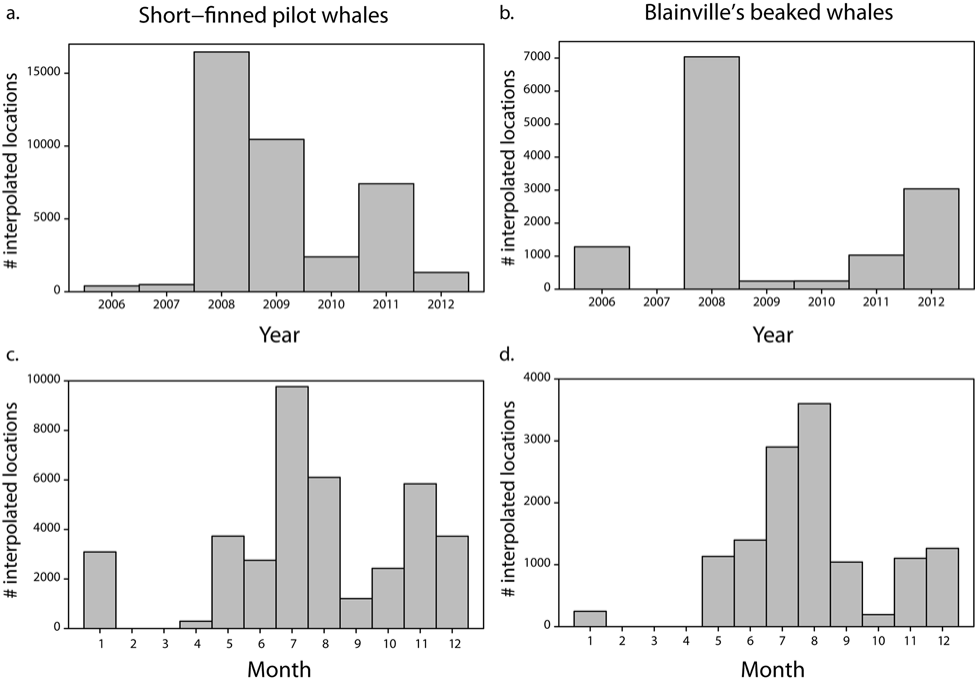
Distribution of whale data. Distribution of interpolated position estimates per year (a-b) or month (c-d) for short-finned pilot whales (a-c) and Blainville’s beaked whales (b-d).

**Fig 2:**
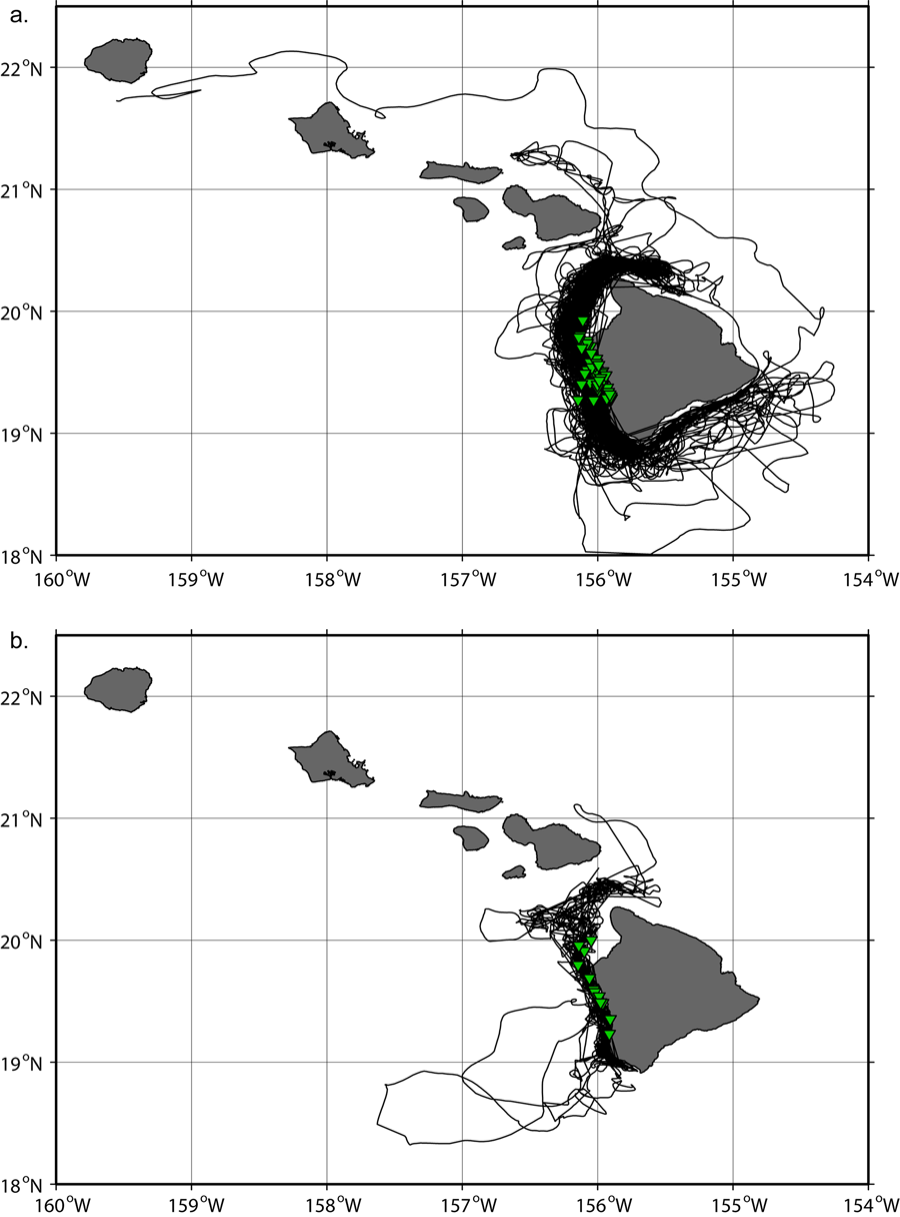
Whale tracks. Maps of filtered and resampled tracks for short-finned pilot whales (a, n = 46) and Blainville’s beaked whales (b, n = 12). Satellite tag deployment locations are represented with green triangles.

**Fig 3:**
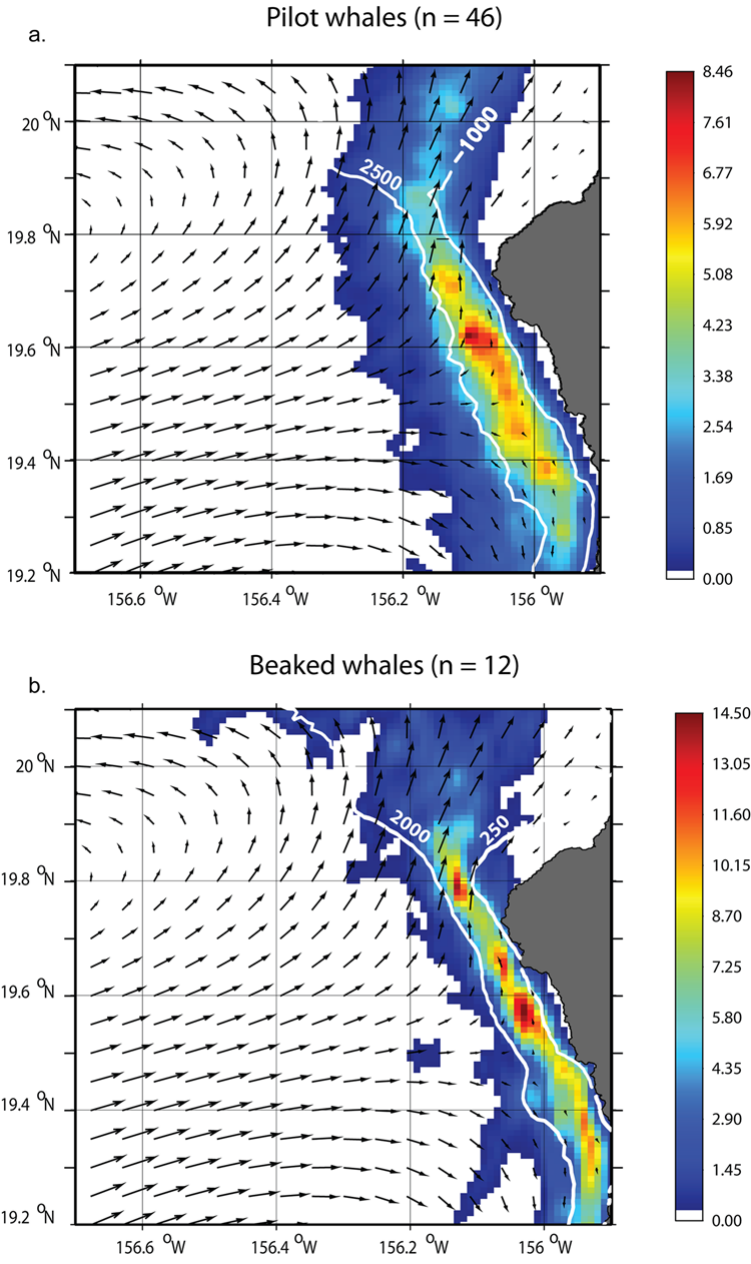
Kernel density estimates. Kernel density estimates of short-finned pilot whales (a) and Blainville’s beaked whales (b) from satellite tag data (color scale) overlaid with bathymetry contours (white lines) and surface current direction (black arrows—the size of the arrow is proportional to current strength). Satellite tag deployment locations are represented with green triangles.

The distribution of resampled tracking data is shown on Fig 1. All tag deployments occurred along the Kona Coast of the Island of Hawai‘i (Table 1, Fig 2). One short-finned pilot whale traveled as far as the southern coast of Kaua‘i, another pilot whale and one Blainville’s beaked whale spent some time off the windward coast of Maui, whereas all other tagged whales remained in the vicinity of Hawai‘i Island or in the ʻAlenuihāhā Channel, between Maui and Hawai‘i Island. The core of the distribution of pilot whales and beaked whales was located between the 1000 and 2500 m isobaths, and the 250 and 2000 m isobaths respectively (Fig 3). Those depth ranges are similar to those found from the analyses of sighting data [4]. Both species were associated with slope waters and the distribution of beaked whales was narrower and in shallower waters than that of pilot whales. For both species, the areas of highest densities generally coincided with areas along the coast where the mean surface currents are relatively weak and almost perpendicular to the coast (Fig 3).

**Fig 4:**
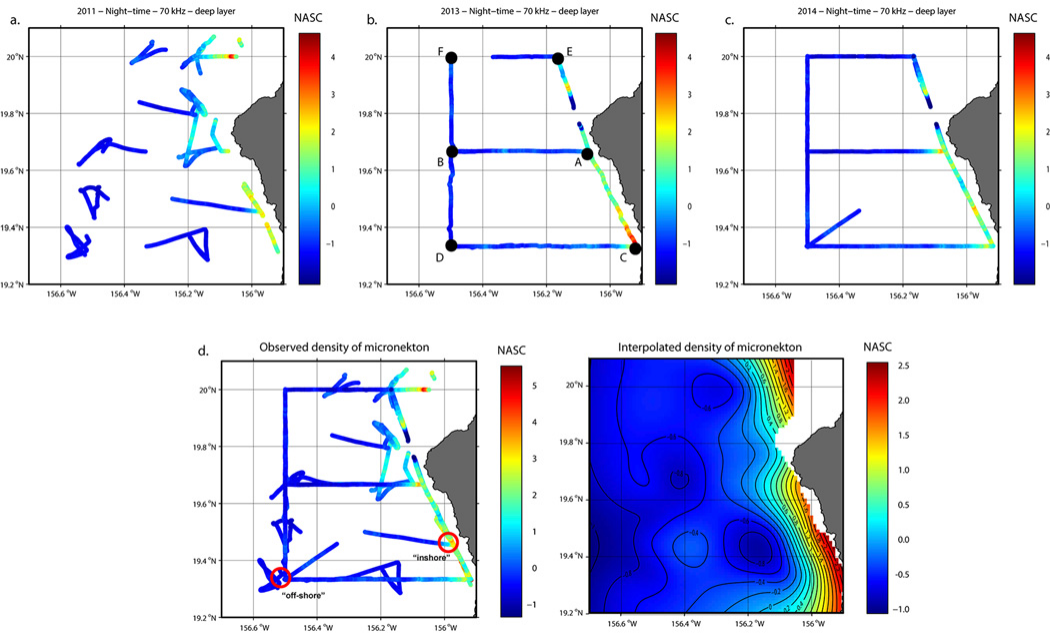
Micronekton distribution. Observed density of micronekton along 2011, 2013, 2014 cruise transects (a–c, respectively), all 3 cruises combined with the “hotspot” and “off-shore” sites circled (d) and corresponding predicted density of micronekton over the study area from the GAM (lon,lat) (e). Values shown here are standardized NASC values for 70 kHz deep night-time. The lettered black dots on Fig 4B represent the 2013 trawling stations.

Standardized observed micronekton densities along ship transects from each of the three cruises, expressed as NASC, show very similar spatial patterns with higher micronekton densities close to the coast and a steep gradient away from shore, with the exception of a patch of higher density in the southeastern corner of the transect in 2013 (Fig 4A–4C). For each micronekton component, standardized data were combined from the 3 cruises (Fig 4D) and the GAM-based extrapolation (Fig 4E) was used in modeling whale distributions.

**Fig 5:**
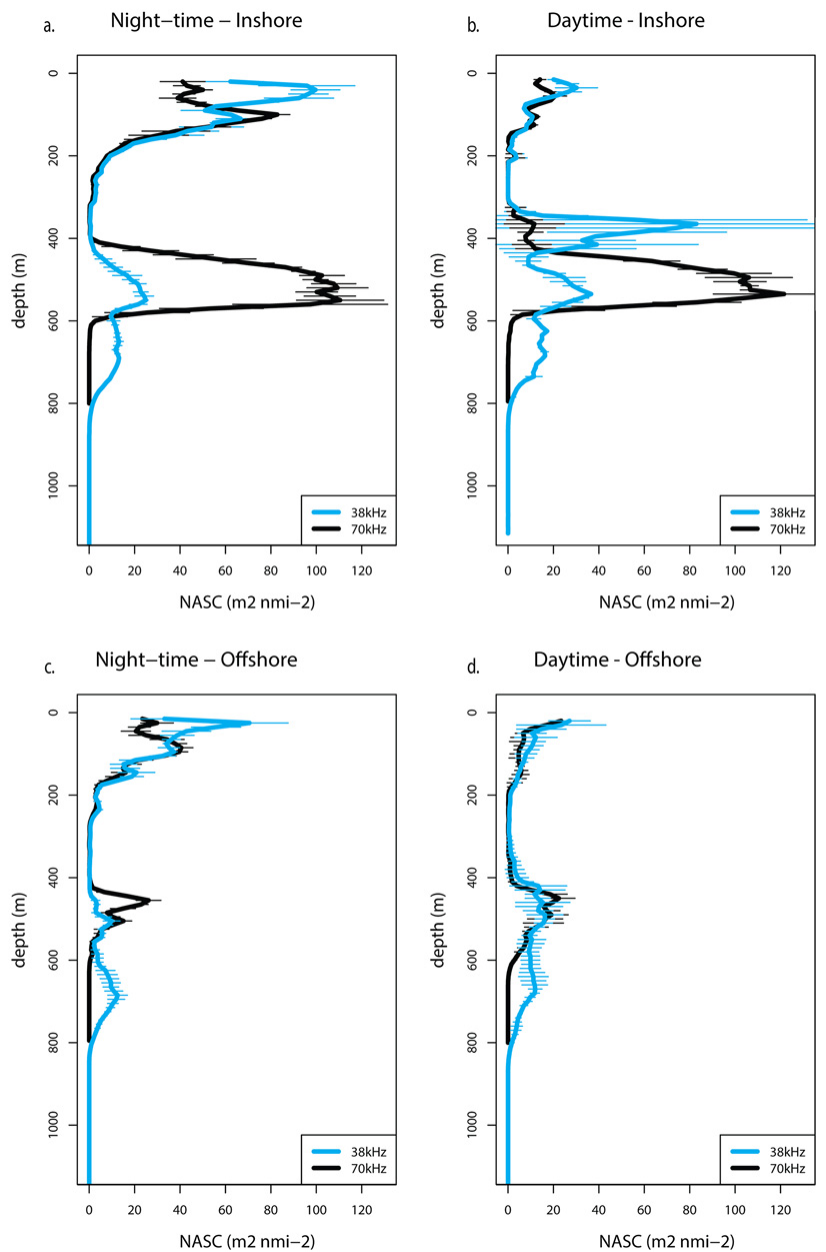
Depth profiles of NASC. Depth profiles of NASC from the 2011 cruise for the 38 kHz (blue lines) and 70 kHz (black lines) transducers at one inshore location (a-b) and one off-shore location (c-d) during the night-time (left) and the daytime (right). See Fig 4D for inshore and offshore locations.

Examination of depth profiles of NASC at various locations shows drastic differences between inshore and off-shore areas (located on Fig 4D), most notably a persistent peak between 400 and 600 m at the 70 kHz frequency, during both night-time and daytime (Fig 5). This pattern is also evident in Fig 4E. The hotspot area also features a peak around 400 m deep at the 38 kHz frequency during the daytime and close to the surface during the night-time as the organisms that scatter relatively strongly at the 38 kHz, mostly micronektonic fish, typically migrate vertically.

**Fig 6:**
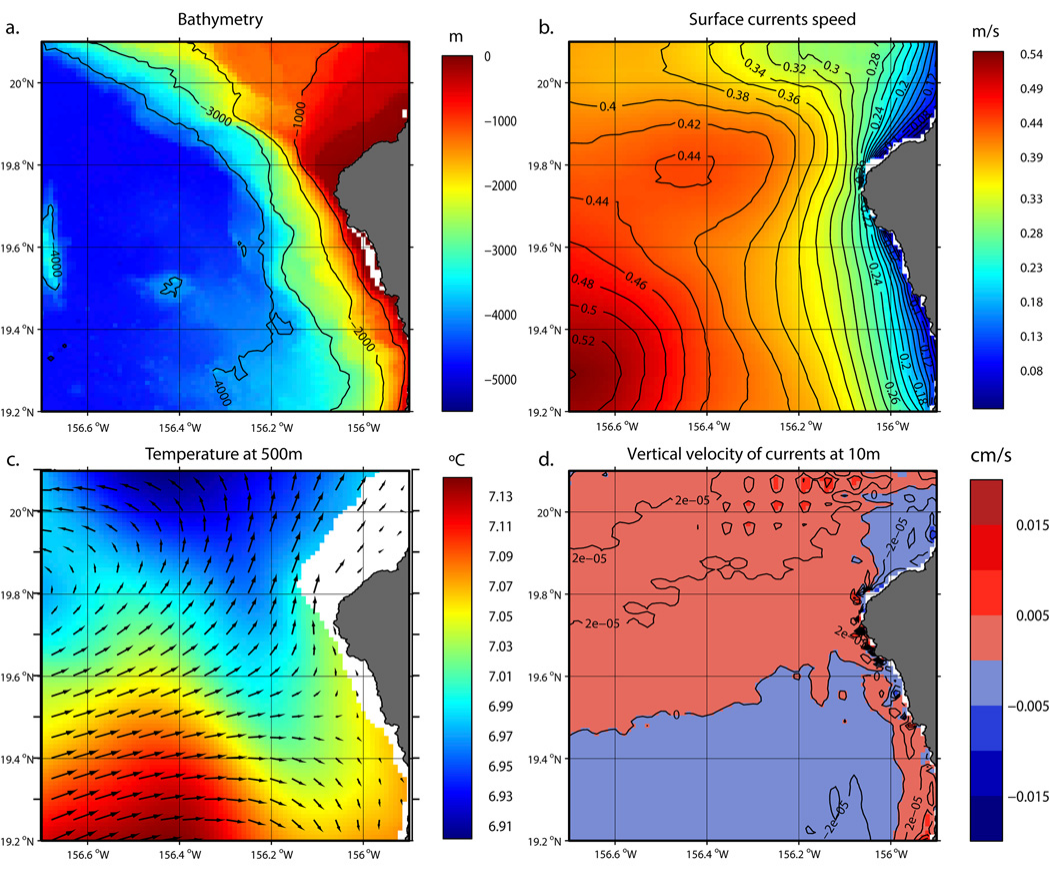
Kona Coast oceanography. Bathymetry (a, m), Currents speed (m/s) at the surface (b), Temperature (°C) at 500 m overlayed with surface currents vectors (c, the white area corresponds to waters shallower than 500 m) and vertical velocity (cm/s) of currents at 10 m (d). See text for data sources.

The southern part of the study area, on the leeward side of the island, was characterized by weak mean surface currents and a very steep bathymetry gradient (Fig 6B and 6A, respectively), with water depths greater than 1000 m accessible close to shore (around 2.5 km). The northern area was characterized by shallower waters (Fig 6A).

Mean temperature at depth exhibits a north-south gradient with slightly warmer waters in the southern half of the study area (Fig 6C). This temperature pattern separates the northern part of the region, which is characterized by an upwelling zone, from the downwelling southern half (south of about 19.7°N, Fig 6D). This pattern of mean current circulation would advect passive organisms away from the coast in the northern part and accumulate them along the coast in the southern part (Fig 6C).

**Fig 7:**
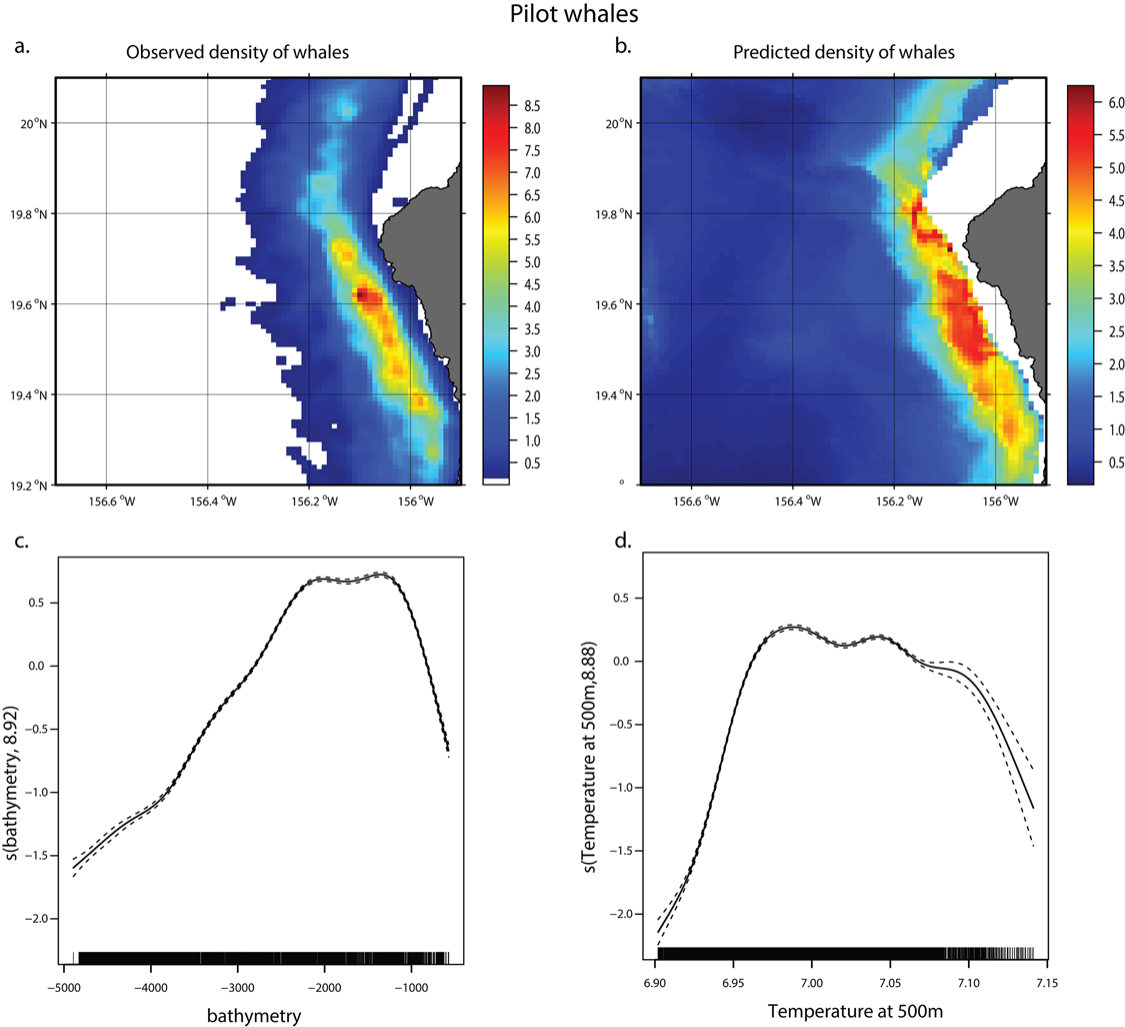
Pilot whale GAMM. Kernel density estimates for short-finned pilot whale (a), predicted short-finned pilot whale density from GAMM (b), smoother from GAMM for bathymetry (c), temperature at 500 m (d). The two scales are not the same to better reflect where the areas of relative highest densities are, according to the density estimates and the model predictions. Dashed lines represent the confidence intervals around the smoothers.

The total amount of deviance explained by the pilot whale GAMM is 31.2% (Table 3). Model predictions match kernel density estimates well, reflecting both the north-south and onshore-off-shore extents of the core distribution of pilot whales as well as its range away from the coast (Fig 7). The best explanatory variable was bathymetry (21.9% of the deviance) followed by temperature at 500 m (8.1%), with highest densities identified between about 1500 and 2500 m and with higher temperatures at depth, reflecting a higher concentration of whales in the downwelling area of the region (Fig 6D).

**Table 3:** Variables in the GAMM for short-finned pilot whales and amount of deviance explained by each.

Variable	deviance explained
**bathymetry**	**21.90%**
**Temperature at 500m**	**8.10%**
Vertical velocity at 200m	1%
**Total**	**31.2%**

When the model was rerun using only 2011 data, the results were very similar, with a total of 28.3% of the deviance explained, with bathymetry explaining 17% and temperature at 500m explaining 9.1%. Similarly the 2011 model predictions matched kernel density estimates well (S1 File).

**Fig 8:**
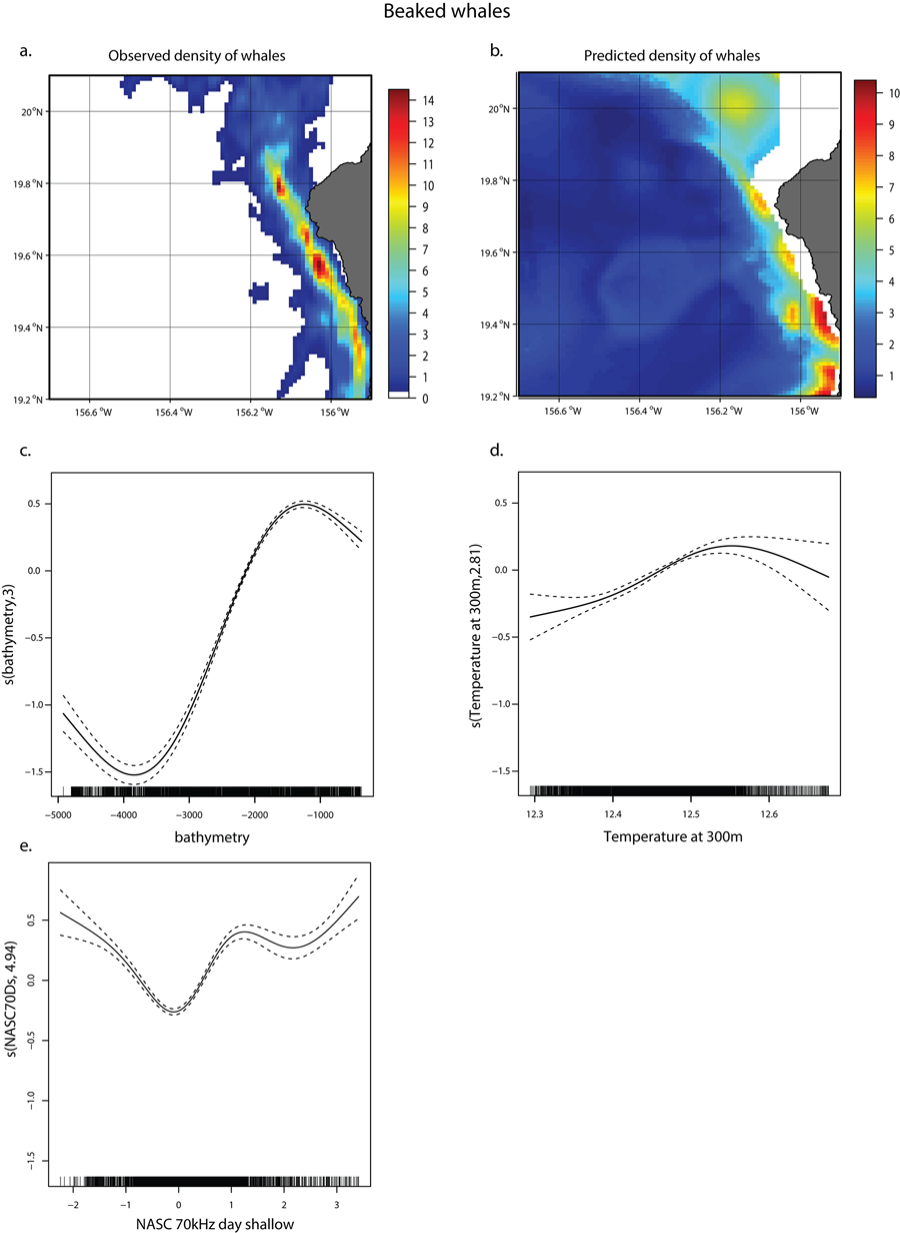
Beaked whale GAMM. Kernel density estimates for Blainville’s beaked whale density (a), predicted Blainville’s beaked whale density from GAMM (b), smoother from GAMM for bathymetry (c), temperature at 300 m (d), micronekton density in the shallow layer observed on the 70 kHz (e). The two scales are not the same to better reflect where the areas of relative highest densities are, according to the density estimates and the model predictions. Dashed lines represent the confidence intervals around the smoothers.

Because the core distribution of Blainville’s beaked whales extends into shallow waters (as shallow as 250-m bottom depth, Fig 3), physical variables for depths greater than 300 m were not considered in order to account for the full extent of their distribution (ie. to avoid selecting variables that would track only the off-shore edge of the beaked whale distribution). For Blainville’s beaked whales, the best model explained 28.5% of the deviance, again with bathymetry as the variable with most explanatory power (24.6%) (Table 4). Temperature at depth was also a factor (1.5%), but to a lesser degree than for pilot whales and the relative biomass of micronekton in the shallow layer observed on the 70 kHz explained 2.4%, with whales predominantly in areas of higher micronekton biomass (Table 4, Fig 8). The predictions matched the distribution of whale density estimates and its narrow extent along the coast (Fig 8).

**Table 4:** Variables in the GAMM for Blainville’s beaked whales and amount of deviance explained by each.

Variable	deviance explained
bathymetry	24.6%
NASC 70kHz Daytime shallow	2.4%
Temperature at 300m	1.5%
**Total**	**28.5%**

Bathymetry and micronekton density are highly correlated and the GAMM selects only bathymetry when both variables are considered. However the effect of micronekton distribution on whale distributions can be addressed with a “trophic-only” single-variable GAMM for each whale species. For both species, the component that explained the maximum amount of deviance in whale distribution was the daytime deep component, observed on the 70 kHz frequency, 15% and 19.7% for beaked and pilot whales, respectively (Table 5), which is about 52% and 63%, respectively, of the deviance explained by each fully-parameterized (environmental and trophic covariates) model (Tables 3 and 4).

**Table 5:** Deviance explained by the biomass of each micronekton component in a single-variable GAMM for Blainville’s beaked whales and short-finned pilot whales.

Variable	Blainville’s Beaked Whales (%)	Short-finned Pilot Whales (%)
38 kHz day shallow	7.7	5.6
38 kHz day deep	6.6	8.7
38 kHz night shallow	6.9	3.1
38 kHz night deep	7.4	17.0
70 kHz day shallow	7.0	3.6
70 kHz day deep	**15.0**	**19.7**
70 kHz night shallow	6.9	1.9
70 kHz night deep	14.4	14.6

**Fig 9:**
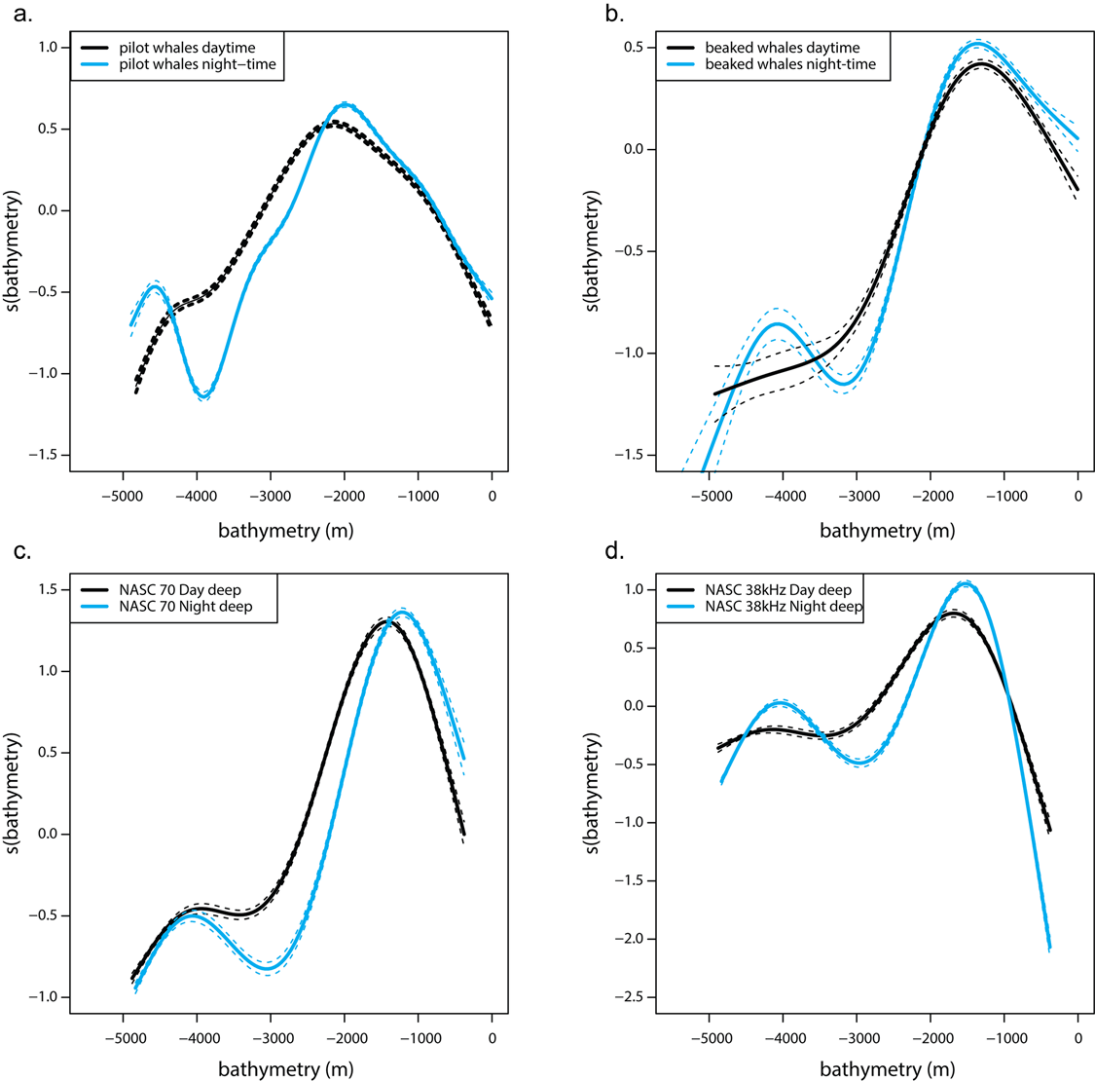
Diel migration. Bathymetry smoothers from GAMMs for short-finned pilot whales (a), and Blainville’s beaked whales (b), and from GAM for 70 kHz NASC (c), 38 kHz NASC (d). Black and blue lines represent daytime and night-time distributions, respectively. Dashed lines represent the confidence intervals around the smoothers.

Short-finned pilot whales and deep micronekton on the 70 kHz frequency exhibited a clear shift of biomass into shallower waters, i.e. more nearshore waters, during the night-time with the bulk of organisms traveling from about 2200 m bottom depth to 2000 m for pilot whales and 1500 m bottom depth to 1100 m for micronekton (Fig 9A and 9C). A similar pattern can be observed for Blainville’s beaked whales and deep micronekton on the 38 kHz, although the shift was less pronounced (Fig 9B and 9D).

On average, *Oplophorus gracilorostris* represented 64% of the caridean shrimps collected during trawling operations at inshore stations compared to 29% at off-shore stations. Similarly, *Abralia trigonura* were present in higher proportions at inshore stations than offshore, with 42% and 26%, respectively, of enoploteuthid squids collected during the 2013 cruise (Table 6).

**Table 6:** Results of trawl samples analysis. With station name (see Fig 4), inshore vs. off-shore location, proportion of *Oplophorus gracilorostris* in caridean shrimps, and proportion of *Abralia trigonura* in enoploteuthid squids.

Station	Inshore/offshore	Mean % of Carideans	Mean % of Enoploteuthids
A	inshore	43	23
B	offshore	23 ± 1	31 ± 11
C	inshore	96 ± 2	70 ± 16
D	offshore	25 ± 10	21 ± 2
E	inshore	42 ± 12	24 ± 1
F	offshore	50	23
Mean	Inshore:	64 ± 32	42 ± 27
Mean	Offshore:	29 ± 14	26 ± 8

## Discussion

We have shown that it is possible to predict the areas of highest use by short-finned pilot whales and Blainville’s beaked whales off the west coast of Hawai‘i Island using bathymetry and climatologies of oceanographic data, primarily temperature at depth, using GAMMs. The best models explained 31% and 29% of the deviance for pilot and beaked whales, respectively (Tables 3 and 4). Additionally, relative micronekton density aggregated over 3 cruises explained 15–20% of the deviance in whale distribution (Table 5) in single-variable GAMMs, with whales following the night-time inshore vs. daytime off-shore migration exhibited by micronekton (Fig 9).

The data we used for this study were all climatologies–whale densities from satellite tag data covering about 5 years from Nov. 2006 to Dec. 2011, standardized relative micronekton biomass from active acoustic data collected during 3 cruises, spanning 3 different years (2011, 2013, 2014) and 2 different seasons (winter and summer), and oceanographic data from a 2-year general circulation model simulation, designed to reproduce the variability and characteristics of the ocean observed in in-situ data, from Apr. 2011 to Jul. 2013. The temporal overlap between those various data sets is minimal but we worked under the assumption that each was representative for our study area. That assumption seems valid for pilot whales, since a rerun of the model using only 2011 data (when all data sets overlap) yielded similar deviance explained, smoothers and predictions (S1 File). However, for beaked whales, for which the sample size is much smaller (n = 12 vs 46), the movement showed by the only tag that was deployed in 2011 is not representative of the overall beaked whale distribution. Thus our results may not be robust across years for beaked whales and a larger sample size would be beneficial.

Despite the fact that the three research cruises occurred at different times of the year, the active acoustic data that was collected showed a persistent pattern of higher micronekton biomass onshore between depths of 400 and 600 m observed on the 70 kHz frequency (Figs 4 and 5). An abundant assemblage of mesopelagic species associated with the island slope distinct from the species found offshore has been documented in the Hawaiian Islands and termed the mesopelagic boundary community, which constitutes a fundamental link for energy and nutrient transfer between nearshore and oceanic ecosystems, while being the most important food for higher trophic levels [42,52–55]. While this community was described in shallower bottom depths, Reid et al. [42] proposed a potential lower mesopelagic boundary component between 700 and 1200 m bottom depth. The layer identified in our data lies over bottom depths around 1000–1400 m and its narrow extent along the slope and near the coast suggests that it may be that lower mesopelagic component of the boundary layer. Analysis of trawl samples collected during the 2013 cruise confirmed that species indicative of the mesopelagic boundary layer were present in those deeper waters occupied by whales, in higher proportions closer to shore than offshore.

Whales and micronekton were found more inshore during the night-time than during the daytime (Fig 9). Mesopelagic boundary layer organisms are capable of undertaking horizontal migrations of at least 11 km roundtrip each night [55] and spinner dolphins (*Stenella longirostris*) in Hawai‘i have been shown to follow the boundary community both horizontally and vertically at night [52]. Our data suggests that pilot whales clearly exhibit the same behavior, and Blainville’s beaked whales as well, albeit to a lesser extent.

However, the active acoustic sampling design offers limited cross-gradient (ie. perpendicular to the bathymetry slope) coverage. More cross-gradient sampling through the areas of highest whale densities would greatly improve the confidence in the interpolated spatial distribution of micronekton, in the extent of the boundary layer, as well as in the relationship identified here between whales and micronekton.

Blainville’s beaked whales prey mostly on fish and small squids [56] and have been shown to prey on both the deeper parts of the deep scattering layer (DSL) and on the benthic boundary layer, with foraging buzzes typically occurring at depths between 700 and 1200 m [21,57]. Our ability to capture those deep targets is limited to about 670 m with the 70 kHz transducer we used for this study, and 1250 m for the 38 kHz. However, Hazen et al. [58] found a strong correlation between micronekton densities and beaked whale foraging effort in the Bahamas, even though the highest micronekton densities were found between 400 and 600 m, as in our study. According to Arranz et al., Blainville’s beaked whales may target prey in the oxygen minimum layer associated with, but deeper than, the bulk of the DSL that are themselves predators of DSL organisms [21]. In the absence of biomass estimates for those prey, it is therefore not surprising to find a reasonably good agreement between whale and micronekton biomass off Hawai‘i Island as well, accounting for more than half of the deviance (15% out of 26%) explained by the best fully-parameterized model. More surprisingly, the density of micronektonic organisms in the shallow layer (0–250 m) was a significant factor in the beaked whale model, although it only explained a small amount of the deviance (2.4%, Table 4).

A stomach from a stranded Blainville’s beaked whale in Hawai‘i contained fish and squids in almost equal proportions, with some of the identified prey (*Serrivomeridae beani*, *Mastigoteuthis famelica*) being fairly mobile non-migrating lower mesopelagic species inhabiting depths 675–960 m (Kristi West, pers. comm., [42]), evidence that beaked whales forage on a mix of slow and fast prey. Similarly, in other areas, short-finned pilot whales have been shown to target fast prey during the daytime. In the Canary Islands, on a typical foraging daytime dive, they start echolocating around 300–400 m deep, searching above the expected depth of prey allowing the whale to search a greater area, then sprinting downward just before buzzing at maximum depths between 538 m and 1019 m [18], likely targeting large squids with high calorific content. Those squids are probably tightly associated with the DSL on which they prey. Two pieces of squids were retrieved at the surface near pilot whale pods that were observed during the 2011 cruise. They were identified as species of *Onykia* and one species of *Onichoteuthid* (William Walker, pers. comm.), with dorsal mantle lengths of 74 cm and 50 cm, respectively.

In conclusion, both species of odontocetes seem to use different foraging strategies between the daytime and the night-time to feed on a mix of fast and slow organisms, all of which are associated with or part of the DSL. Most likely, those prey organisms forage on the lower mesopelagic boundary community inhabiting the slope waters of the Island of Hawai‘i, which explains why bathymetry appears as the best predictor of whale density. Thus, our results suggest that off Kona, and possibly around other Hawaiian islands, the deep mesopelagic boundary community may be a key part of a food web that supports insular cetacean populations.

Additionally, and to a lesser extent, the general pattern of ocean currents in the area promotes organism retention and potentially increases the base of the food web in the southern part of the Kona Coast, both contributing to an enhanced density of the mesopelagic boundary community.

## Supporting Information

S1 File Examination of 2011 data. (PDF)
