# Supplementary material for: Characterizing a Foraging Hotspot for Short-Finned Pilot Whales and Blainville’s Beaked Whales Located off the West Side of Hawai‘i Island by Using Tagging and Oceanographic Data
Source: PLoS One. 2015 Nov 25;10(11):e0142628. doi: 10.1371/journal.pone.0142628 (PMC4659615; doi:10.1371/journal.pone.0142628)

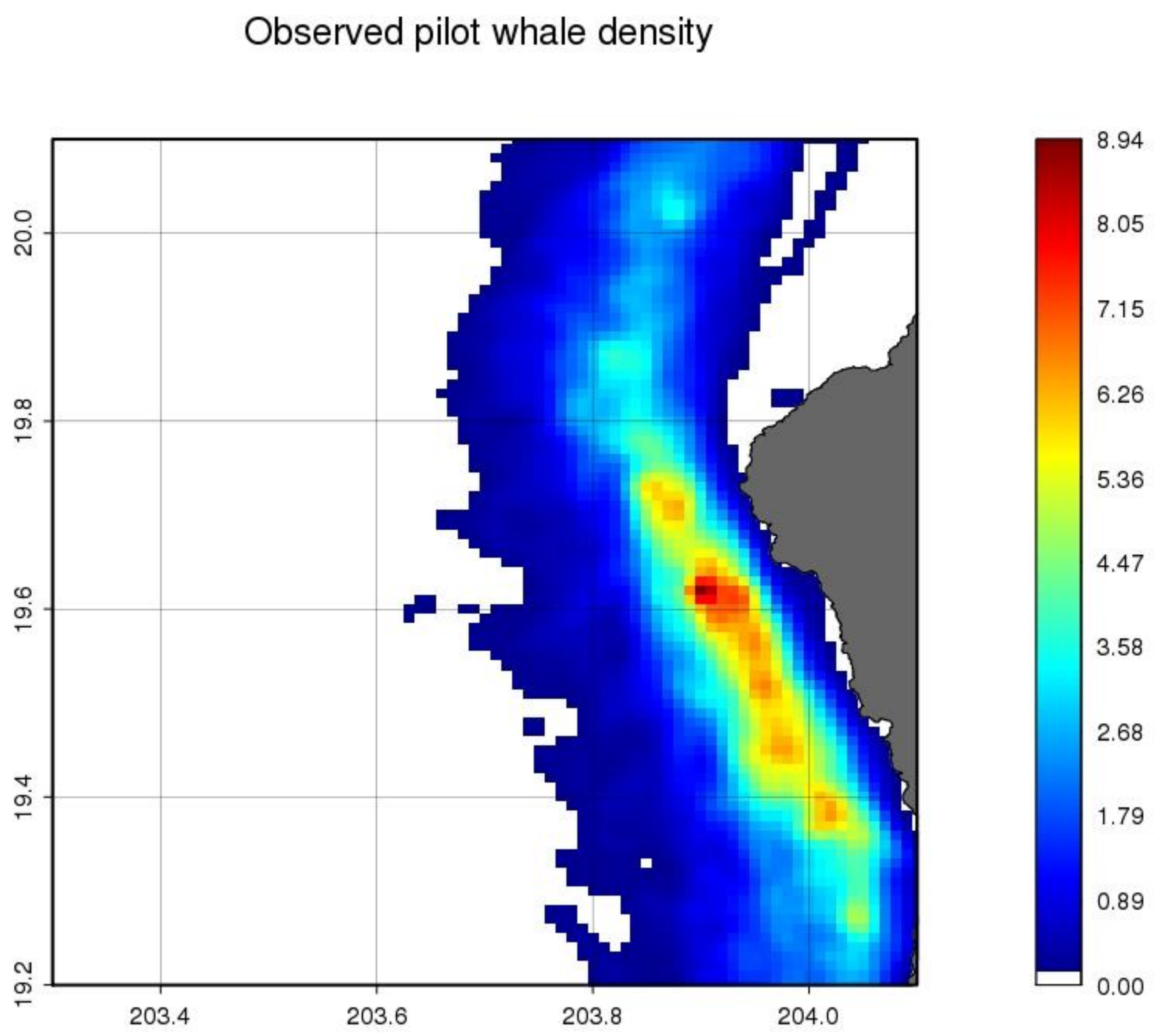

Observed pilot whale density
20.0
19.8
19.6
19.4
19.2
203.4
203.6
203.8
204.0
8.94
8.05
7.15
6.26
5.36
4.47
3.58
2.68
1.79
0.89
0.00

Observed pilot whale density - tags at sea for > 10 days

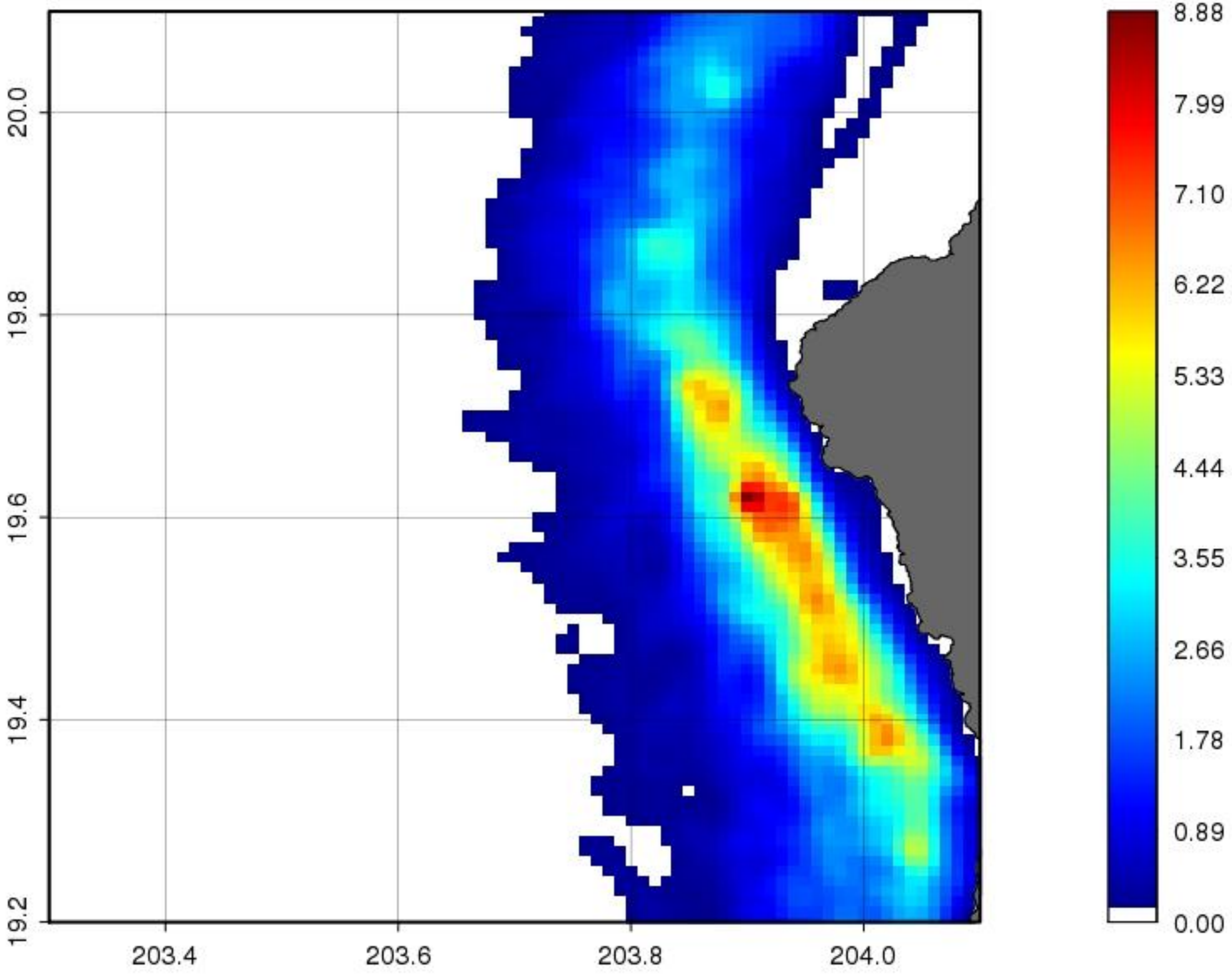

Observed pilot whale density - 2011

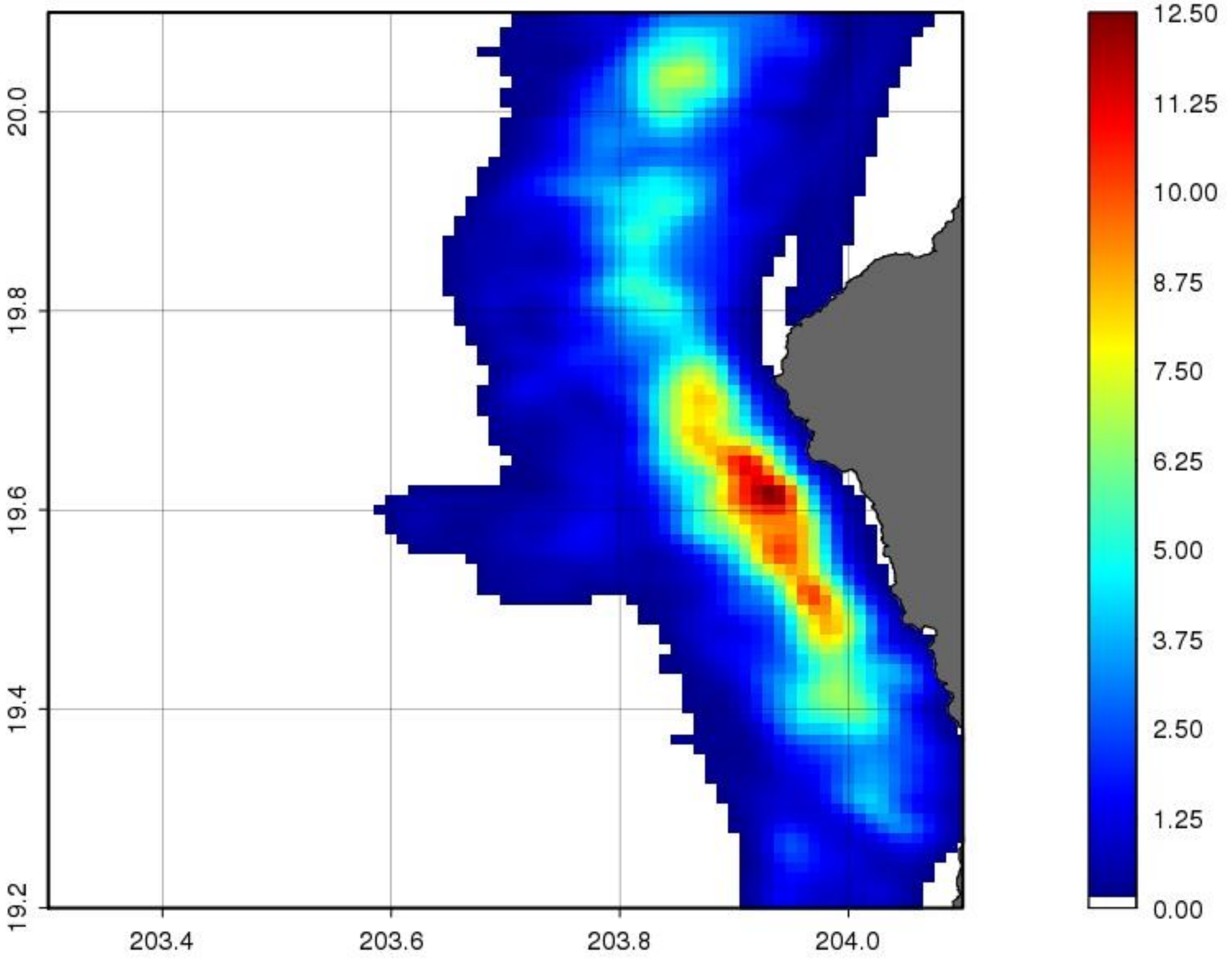

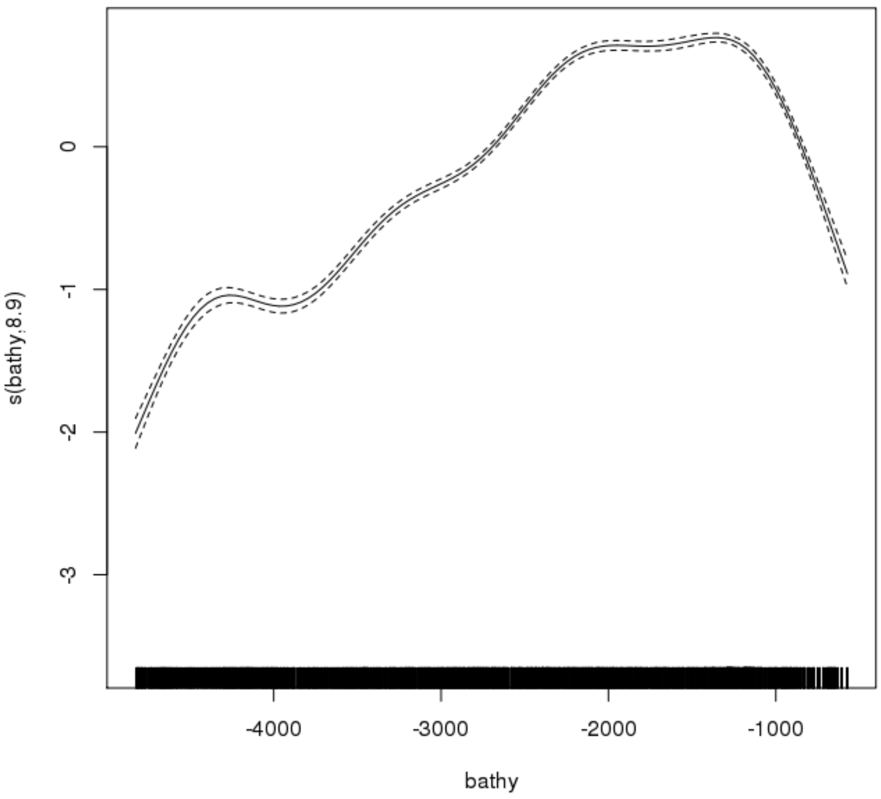

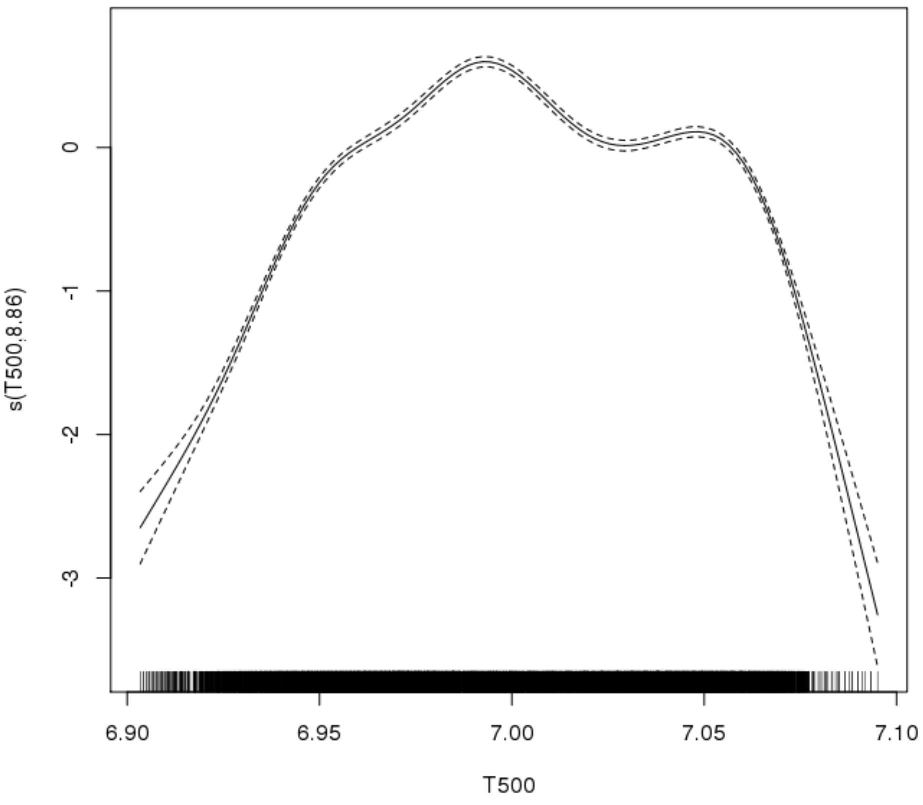

variable	deviance explained
bathymetry	17.0%
Temperature at 500m	9.1%
Vertical velocity at 200m	2.2%
Total	28.3%

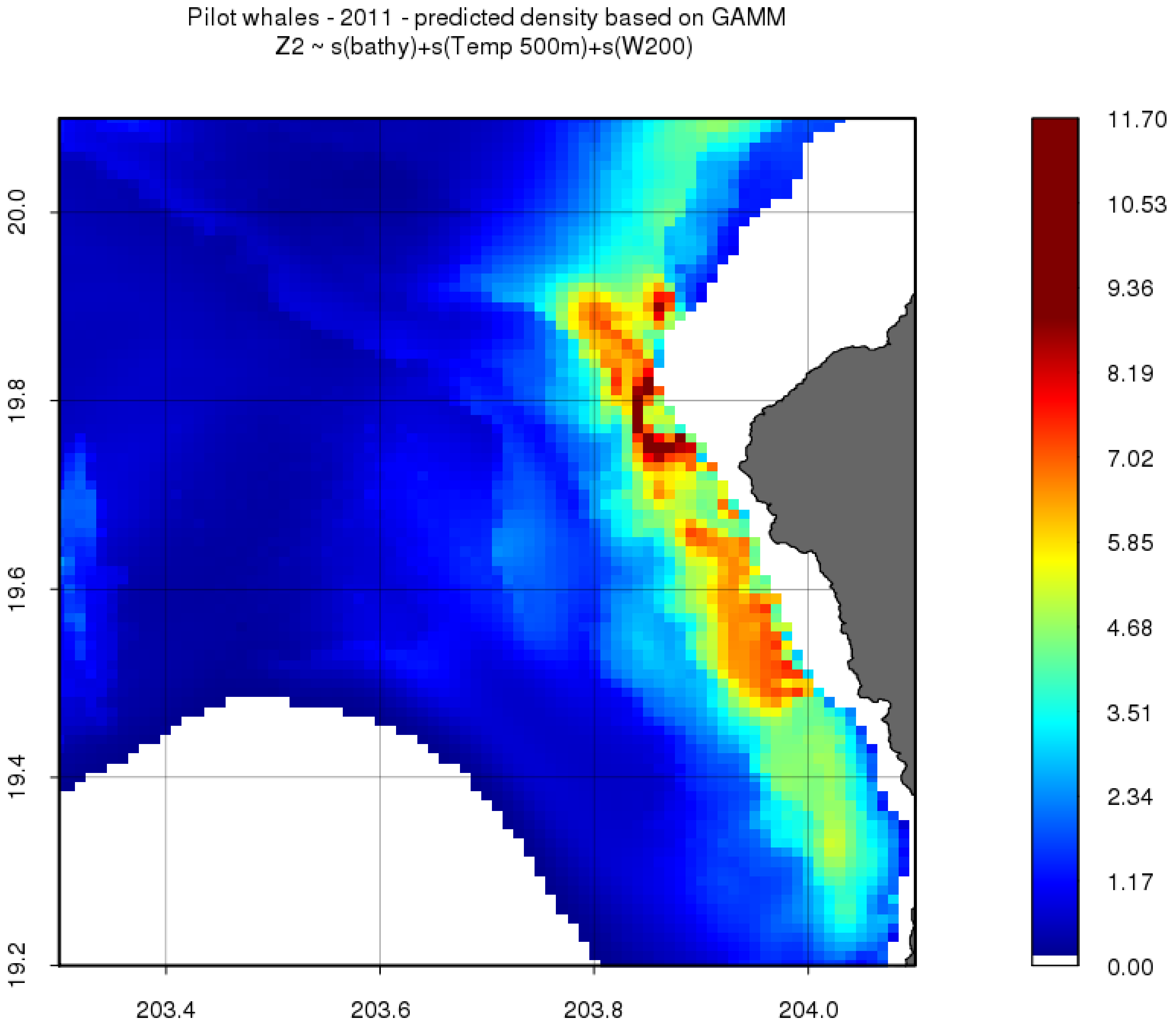

Pilot whales - 2011 - predicted density based on GAMM
Z2 ~ s(bathy)+s(Temp 500m)+s(W200)
20.0
19.8
19.6
19.4
19.2
203.4
203.6
203.8
204.0
11.70
10.53
9.36
8.19
7.02
5.85
4.68
3.51
2.34
1.17
0.00

Observed beaked whale density

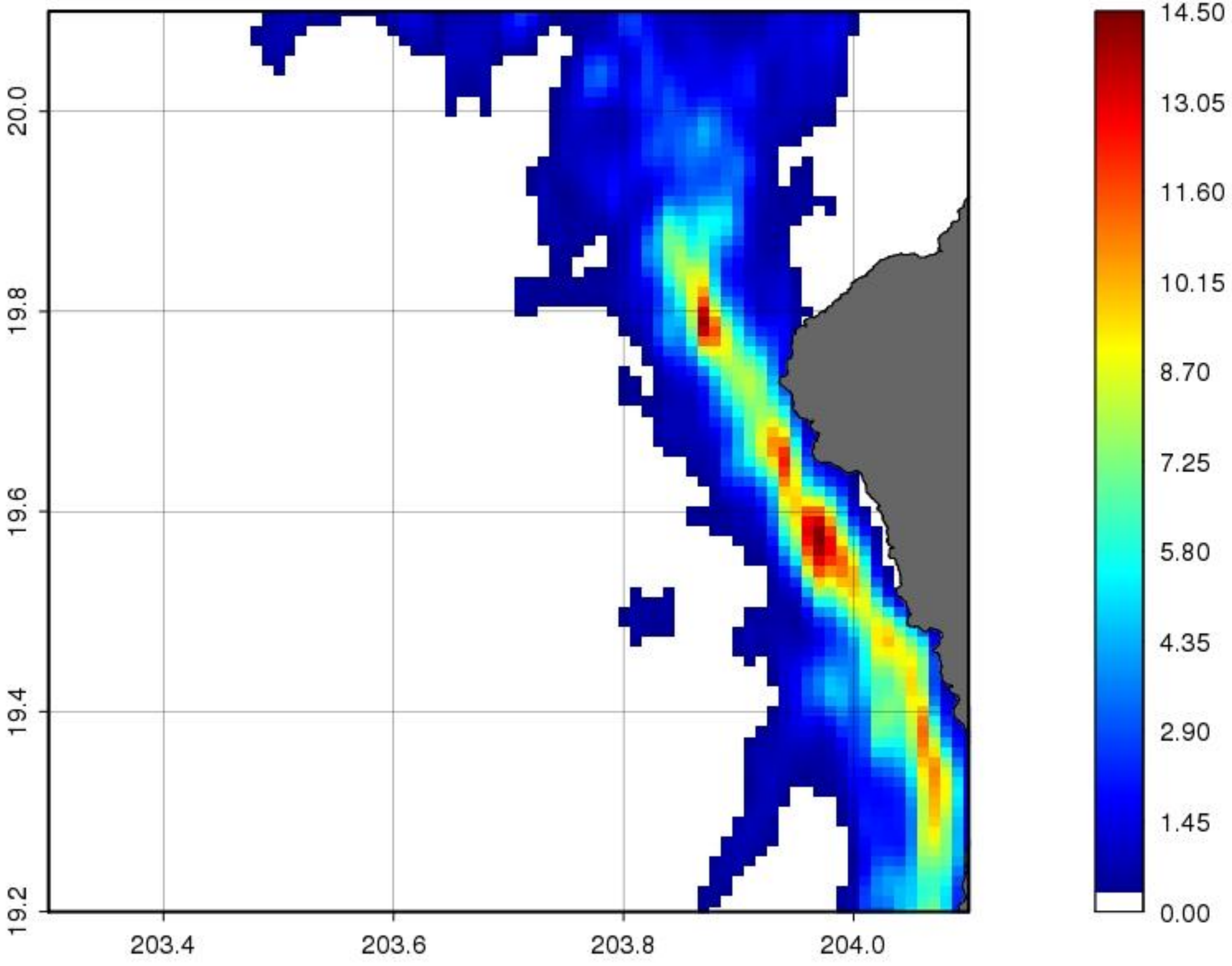

Observed beaked whale density - 2011

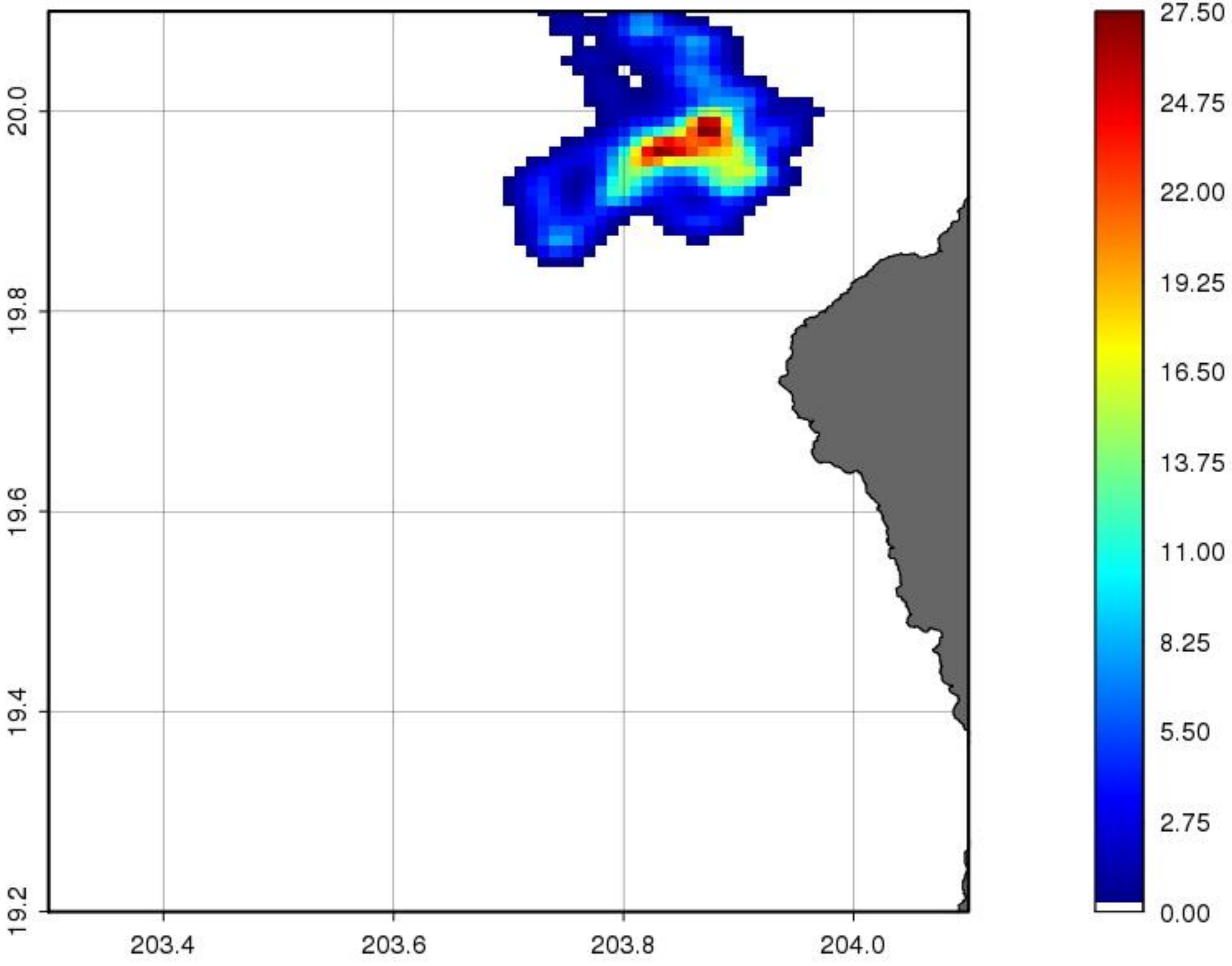

Supplement: S1 File — (PDF) [file pone.0142628.s001.pdf]
